# Dysregulation of circulating T follicular helper cell subsets and their potential role in the pathogenesis of syphilis

**DOI:** 10.3389/fimmu.2023.1264508

**Published:** 2023-10-12

**Authors:** Fuping Shen, Yuhuan Shen, Yuni Xu, Jiwei Zhao, Zhao Zhao, Jinlin Liu, Yumei Ge

**Affiliations:** ^1^ Laboratory Medicine Center, Department of Clinical Laboratory, Zhejiang Provincial People’s Hospital (Affiliated People’s Hospital), Hangzhou Medical College, Hangzhou, Zhejiang, China; ^2^ School of Medical Technology and Information Engineering, Zhejiang Chinese Medical University, Hangzhou, Zhejiang, China; ^3^ Department of Laboratory Medicine, The Second Affiliated Hospital of Hainan Medical University, Haikou, China; ^4^ Department of Laboratory Medicine, Nanjing Lishui District Hospital of Traditional Chinese Medicine, Nanjing, Jiangsu, China; ^5^ Department of Clinical Laboratory, South China Hospital, Medical School, Shenzhen University, Shenzhen, China

**Keywords:** Tfh17 cells, ICOS + Tfh, effector memory Tfh, secondary syphilis, latent syphilis

## Abstract

**Introduction:**

The role of the host immune response could be critical in the development of Treponema pallidum (Tp) infection in individuals with latent syphilis. This study aims to investigate the alterations in T follicular helper T (Tfh) cell balance among patients with secondary syphilis and latent syphilis.

**Methods:**

30 healthy controls (HCs), 24 secondary syphilis patients and 41 latent syphilis patients were enrolled. The percentages of total Tfh, ICOS^+^ Tfh, PD-1^+ ^Tfh, resting Tfh, effector Tfh, naïve Tfh, effector memory Tfh, central memory Tfh,Tfh1, Tfh2, and Tfh17 cells in the peripheral blood were all determined by flow cytometry.

**Results:**

The percentage of total Tfh cells was significantly higher in secondary syphilis patients compared to HCs across various subsets, including ICOS^+ ^Tfh, PD-1^+^ Tfh, resting Tfh, effector Tfh, naïve Tfh, effector memory Tfh, central memory Tfh, Tfh1, Tfh2, and Tfh17 cells. However, only the percentages of ICOS^+ ^Tfh and effector memory Tfh cells showed significant increases in secondary syphilis patients and decreases in latent syphilis patients. Furthermore, the PD-1^+ ^Tfh cells, central memory Tfh cells, and Tfh2 cells showed significant increases in latent syphilis patients, whereas naïve Tfh cells and Tfh1 cells exhibited significant decreases in secondary syphilis patients when compared to the HCs. However, no significant change was found in resting Tfh and effector Tfh in HCs and secondary syphilis patients or latent syphilis patients.

**Discussion:**

Dysregulated ICOS^+ ^Tfh or effector memory Tfh cells may play an important role in immune evasion in latent syphilis patients.

## Introduction

Syphilis is a sexually transmitted infection that is caused by the spirochetal bacterium *Treponema pallidum* (*Tp*) (1). Untreated syphilis can progress through the following four stages: primary, secondary, latent, and tertiary stages. As a multistage disease, syphilis starts with the primary lesion or chancre (2), while secondary syphilis results from disseminated *Tp* infection despite high titers of anti-*Tp* antibodies (1). Latent syphilis is diagnosed based on positive Tp antibodies but with no clinical symptoms according to the 2020 European guideline on the management of syphilis (specifically stated in the “Materials and Methods” section) (3). *Tp* may seed the blood stream intermittently during this stage and reactivate when human immunity is weak (4–6). Because there are no clinical symptoms, latent syphilis patients are often overlooked and have delayed treatment, which can lead to neurological or other organ damage. Therefore, the control or prevention of latent *Tp* infection is an important issue in eradicating syphilis.

T cell-mediated immunity is critical for anti-*Tp* host defense. However, the exact cellular subset responsible for *Tp* clearance has not been well elucidated. CD4^+^ Tcells are believed to eradicate *Tp* through IFN-γ production in syphilitic lesions (7).Other studies suggest that activated CD8^+^ Tcells play an important role in local *Tp* clearance (8, 9). Furthermore, although a strong immune response can result in effective local clearance of *Tp*, *Tp* can persist in many tissues without causing clinical symptoms in the latent stage. Thus, fully elucidating the immune evasion mechanism of *Tp* in latent syphilis patients is essential to effectively eradicate *Tp* in the human body.

T follicular helper (Tfh) cells are a specialized subset of CD4^+^ T cells that support B cells in germinal centers and are necessary for the differentiation of B cells into memory B cells, plasma cells, and germinal center formation (10, 11). In healthy humans, Tfh cells typically constitute a small proportion of the total T cell population, accounting for approximately 1-3% of CD4+ T cells. However, this subset can promote B cell differentiation, and immunoglobulin secretion and help maintain humoral immunity (12, 13). In bacterial infections, Tfh can orchestrate humoral immunity against bacterial pathogens (14, 15) including supporting the B cell differentiation and promoting high-affinity antibody maturation and production, which in turn can neutralize bacteria, aid in bacterial clearance, and foster long-term immune memory. Moreover, Tfh cells play a pivotal role in enabling the immune system to effectively recognize and eliminate bacterial threats by promoting antibody diversity, affinity maturation, and memory formation. Furthermore, during viral infection, virus-specific Tfh cells can also activate the B cells to increase antibody production (16). Similar to CD8^+^ T cells, virus-specific Th1 cells gradually decrease, but Tfh cell differentiation is greatly expanded during chronic infection (16). This emphasis on virus-specific Tfh cells is pivotal for the continued maturation or adaptation of the antibody response, leading to the neutralizing antibodies which can eventually fight against the established chronic infection (16).

However, the roles of Tfh cells in syphilis are currently unknown. In this study, we examined changes in cellular homeostasis in Tfh cells in secondary and latent syphilitic infection patients and aimed to illuminate the potential contributions of Tfh cells to the pathogenesis of syphilis and gain insights into their role in modulating humoral immune responses during the infection. A comprehensive understanding of the dynamic shifts within Tfh cell subsets and their potential associations with disease progression may engender novel avenues for the understanding of the immune response of syphilis.Materials and methods

### Patients

In total, 65 serologically confirmed syphilis patients were enrolled (**Table 1**). The control group included 30 healthy gender-matched individuals who had undergone physical examinations in our hospital. Secondary syphilitic infection patients and latent syphilis patients were diagnosed based on TRUST and TPPA results and clinical history (17). The criteria for the diagnosis of different syphilis stages have been previously reported (3). According to the 2020 European guideline on the management of syphilis, the diagnosis and classification of latent syphilis were described as positive serological tests for syphilis with no clinical evidence of treponemal infection and one of the following conditions: 1) a negative syphilis serology ≥1 year or an unknown duration of syphilis diagnosis, 2) a fourfold or greater increase in nontreponemal antibody titers ≥1 year or unknown duration of previous testing, or 3) unequivocal evidence that the disease was acquired ≥1 year or an unknown duration (on the basis of clinical signs in patients and partners). Additionally, HIV, HBV, and/or HCV positive patients were excluded from this study. All blood collected was for routine blood tests, and the remaining blood was used for this study, which was in accordance with the ethical standards of the Declaration of Helsinki(approval number: 2019KY027) and was approved by the ethical committee of Zhejiang Provincial People’s Hospital.

**Table 1:** Baseline characteristics of the total study population.

	Healthy controls	secondary syphilis	latent syphilis
No. of subjects	30	24	41
Age, years			
Median (range)	33(22-56) 33.03±9.19	55(27-87) 55.00±18.50	54(23-84) 54.05±14.83
45 ≤45	5(16.67%) 25(83.33%)	16(66.67%) 8(33.33%)	27(65.85%) 14(34.15%)
Gender			
Male	11	10	23
Female	19	14	18
HBV Positive	N	N	N
HCV Positive	N	N	N
HIV Positive TRUST(≤1:16) TRUST(1:16) TPPA	N N N N	N 7(29.17%) 17(70.83%) 3.12±0.58	N N N 2.56±0.99

TRUST, toluidine red unhealed serum test; TPPA, treponema pallidum particle assay; HBV, hepatitis B Virus; HCV, hepatitis C Virus; HIV, human immunodeficiency virus; N, negative.

### Blood collection

Venous blood samples were collected from all participants, and after centrifugation at 3000 rpm for 10 minutes, serum was separated for serological testing. The specific anticoagulant used for blood collection was Ethylenediaminetetraacetic Acid (EDTA) and the concentration of EDTA used for anticoagulation in our blood collection tubes was 1.5 milligrams per milliliter (mg/mL) of blood.

### Flow cytometry

Peripheral blood mononuclear cell separation and Tfh cell surface markers incubation were performed as previously described (12). Briefly, the CD4, CD25, CD127, CXCR5, CCR7, CCR6, PD-1, CXCR3, ICOS, CCR7, and CD45RO antibodies were used in this study (**Table 2**), similar to our previous research report (18). Cells stained with separate antibodies were defined as Tfh cells (CD4^+^CXCR5^+^CD25^low^CD127^intermediate-high^), Tfh1 cells (CXCR3^+^Tfh), Tfh2 cells (CXCR3^−^CCR6^−^Tfh), Tfh17 cells(CCR6^+^Tfh),central memory (CD45RO^+^CCR7^+^)Tfh cells, effector memory (CD45RO^+^CCR7^-^)Tfh cells, effector(CCR7^low^PD-1^high^)Tfhcells, naïve (CD45RO^−^CCR7^+^)Tfh cells,resting(CCR7^high^PD-1^low^) Tfhcells and T follicular regulatory (Tfr) cells (CD4^+^CXCR5^+^CD25^intermediate-high^CD127^low^). Samples were analyzed by flow cytometry (Navios, Beckman Coulter, model number: AV17226), and the results were analyzed by Kaluza software (Beckman Coulter, version number: 2.2.1).

**Table 2:** Antibody panel used for flow cytometric analysis of PBMC and their subpopulations.

Antibody-fluorochrome	Clone	Vendors	Catalog number
Alexa Fluor 647 Mouse anti-Human CD127	HIL-7R-M21	BD Pharmingen	558598
APC-R700 Mouse Anti-Human CD25	2A3	BD Horizon	565106
BV421 Rat Anti-Human CXCR5(CD185)	RF8B2	BD Horizon	562747
BV510 Mouse Anti-Human CD4	SK3	BD Horizon	562970
BB515 Rat Anti-Human CCR7(CD197)	3D12	BD Horizon	565869
PE Mouse Anti-Human CD196(CCR6)	11A9	BD Pharmingen	551773
BB700 Mouse Anti-Human CD279(PD-1)	EH12.1	BD Horizon	566460
PE-Cy^TM^7 Mouse Anti-Human CD183	1C6/CXCR3	BD Pharmingen	560831
BB515 Mouse Anti-Human CD278(ICOS)	DX29	BD Horizon	564549
BB700 Rat Anti-Human CCR7(CD197) PE-Cy^TM^7 Mouse Anti-Human CD45RO	3D12 UCHL1	BD Horizon BD Pharmingen	566437 560608

PBMC, human peripheral blood mononuclear cells; APC, allophycocyanin; PE, phycoerythrin.

### Clinical parameters

Venous blood samples were collected from all participants, placed in tubes, and centrifuged at 3000 rpm for 10 min. In detail, the toluidine red unheated serum test (TRUST; RongshengBiotech, China) was used in combination with treponemal pallidum particle agglutination (SERODIA-TP.PA; FUJIREBIO Inc,Japan) for the serological detection of syphilis patients. Our laboratory is certified according to ISO 15189 standards, and the quality of data was validated throughout the study period using internal quality control (IQC) procedures and participation in an External Quality Assessment (EQA) scheme.

### Statistical analysis

GraphPad Prism 5.0.1 software was used for statistical analysis. Initially, an evaluation of the normality of the data distribution was conducted. Non-conformity to a normal distribution necessitated the application of a non-parametric analysis, specifically employing the Kruskal-Wallis test for comparisons across multiple groups. Conversely, in cases where the data exhibited a reasonably normal distribution, meeting parametric assumptions, an assessment of homogeneity of variances was pursued. Homogeneity of variances was rigorously appraised using the Brown-Forsythe test, particularly in instances where variances displayed a lack of uniformity. Subsequently, when both the normality and homogeneity of variances assumptions were satisfied, indicative of the appropriateness of parametric analysis, a one-way analysis of variance (ANOVA) was conducted. The results of these analyses, including *p*-values and relevant statistics, were presented in the figures within the manuscript.Quantitative data are presented as mean ± standard deviation (SD). *p<0.05, **p<0.01, and ***p<0.001 were considered statistically significant.

## Results

### The percentage of CD4^+^CXCR5^+^CD25^low^CD127^intermediate-high^Tfh cells significantly increased in secondary syphilis patients

**Figure 1:**
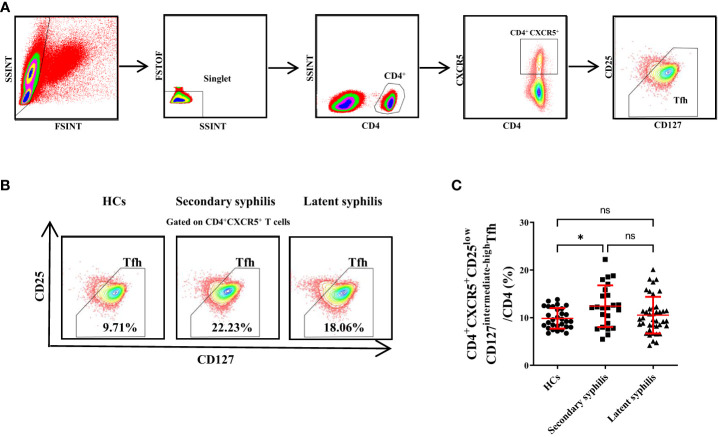
The percentages of CD4^+^CXCR5^+^CD25^low^CD127^intermediate-high^Tfhcellsin total CD3^+^CD4^+^ T cells were significantly increased in secondary syphilis patients. **(A)** The gating criteria for CD4^+^CXCR5^+^CD25^low^CD127^intermediate-high^Tfh cells from the peripheral blood are shown. **(B)** Representative plots of the percentage of CD4^+^CXCR5^+^CD25^low^CD127^intermediate-high^Tfh cell in total Tfh in HCs (left), secondary syphilis patients (middle), and latent syphilis patients (right) are shown. **(C)** The percentage of CD4^+^CXCR5^+^CD25^low^CD127^intermediate-high^Tfh cells in total CD3^+^CD4^+^ T cells was compared between the 30 HCs, 24 secondary syphilis, and 41 latent syphilis patients. *p<0.05, One-way ANOVA test. ns, not significant.

Peripheral blood samples from 30 HCs, 24 secondary syphilis infection patients and 41 latent syphilis patients were analyzed by flow cytometry, and CD4^+^CXCR5^+^CD25^low^CD127^intermediate-high^Tfh cells were identified (**Figure 1A**). Then, the CD4^+^CXCR5^+^CD25^low^CD127^intermediate-high^Tfh cells were defined as the total Tfh cells in our subsequent investigations. Representative fluorescence-activated cell sorting profiles indicating total Tfh (CD4^+^CXCR5^+^CD25^low^CD127^intermediate-high^). Plots were pregated on CD4^+^CXCR5^+^ cells and examined according to the levels of CD25 and CD127. The numbers indicate the proportion of cells in the gate (**Figure 1B**). We found that the percentage of total Tfh cells (CD3CD4CXCR5) to total T cells (CD3CD4) was significantly increased in secondary syphilis infection patients compared with HCs(*p*=0.0266) (**Figure 1C**). However, there was no significant difference in latent syphilis patients when compared to HCs and secondary syphilis patients (**Figure 1C**).

### The percentage of ICOS^+^Tfh cells was significantly different in both secondary and latent syphilis patients

**Figure 2:**
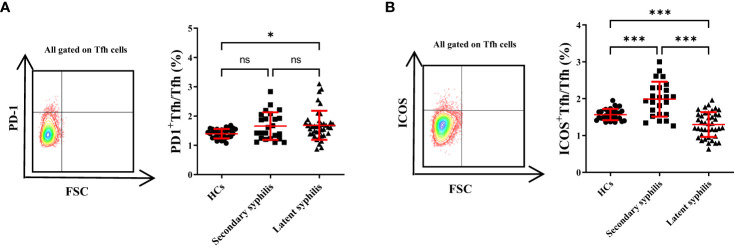
The percentage of ICOS^+^Tfh cells in total Tfh cells showed a significant increase, whereas PD-1^+^Tfh cells did not exhibit significant changes. **(A)** Representative plots of PD-1^+^Tfh and ICOS^+^Tfh cell percentages (upper-right quadrant) in the peripheral blood of HCs (left), secondary syphilis patients (middle),and latent syphilis patients (right)are shown. **(B)** The percentages ofPD-1^+^Tfh and ICOS^+^Tfh cells in total Tfh cells were compared between the 30 HCs, 24 secondary syphilis patients, and 41 latent syphilis patients. **p<0.01, ***p<0.001, one-way ANOVA test. ns, not significant.

Tfh cells typically express CXCR5, PD-1, and ICOS, and previous studies showed that upregulation of these markers in Tfhcells was associated with abnormally high autoantibody titers in autoimmune patients (19). Thus, the percentages of ICOS^+^ and PD-1^+^Tfh cell subsets to total Tfh cells were investigated in secondary syphilis and latent syphilis patients. Among them, the PD-1^+^Tfh cell subset displayed a notable difference between the healthy and latent groups, with no significant distinctions evident between the healthy and secondary, or the secondary and latent groups (**Figure 2A**). Conversely, the ICOS^+^Tfh cell subset exhibited significant differences across all three group comparisons (HCs vs. Secondary syphilis patients p = <0.0001, HCs vs. Latent syphilis patients p = 0.0035, Secondary syphilis patients vs. Latent syphilis patients *p* = <0.0001) (**Figure 2B**).

### The percentages of CCR7^high^ PD-1^low^restingTfhand CCR7^low^PD-1^high^ effector Tfh cells were not significantly different in secondary and latent syphilis patients

**Figure 3:**
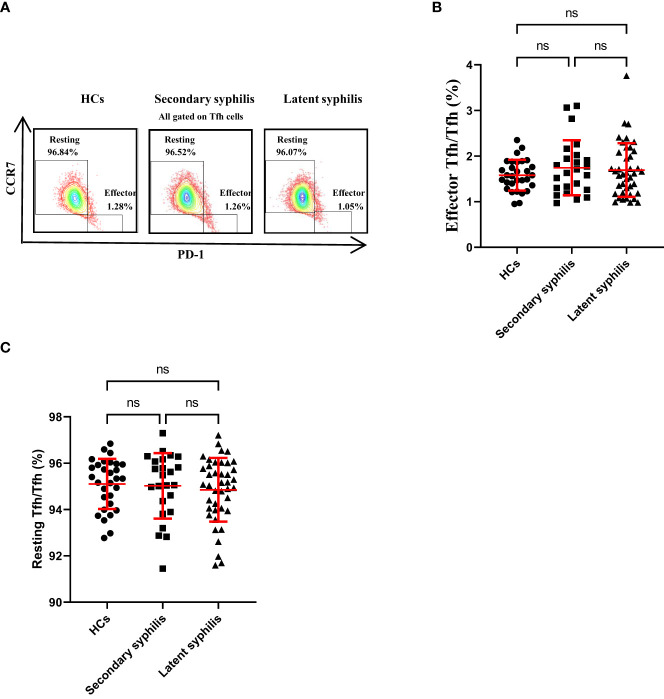
The percentages of CCR7^high^ PD-1^low^restingTfh and CCR7^low^PD-1^high^ effector Tfh cells in total Tfh cells were not significantly different in syphilis patients. **(A)** Representative plots of CCR7^low^PD-1^high^ effector Tfh cell percentages(lower-right quadrant) and CCR7^high^ PD-1^low^resting Tfh cell percentages (upper-left quadrant) within the Tfh cell population in the peripheral blood ofHCs (left), secondary syphilis patients (middle) and latent syphilis patients (right) are shown. The percentages of **(B)** effector Tfh cells and **(C)** resting Tfh cells were compared between the 30 HCs, 24 secondary syphilis spatients, and 41 latent syphilis patients. One-way ANOVA test. ns, significant.

Previously, He (20) found that the CCR7^low^PD-1^high^ subset has a partial effector phenotype and that the CCR7^high^PD-1^low^ subset has a resting phenotype in humans and a circulated CCR7^low^PD-1^high^ subset, indicating an active Tfh program. Results revealed no significant differences in the percentages of these Tfh subsets to total Tfh cells among the HCs, secondary syphilis infection, and latent syphilis patients (**Figures 3A–C**), indicating that these Tfh subsets may not participate in the pathogenesis of syphilis.

### The percentage of effector memory Tfh cells was significantly different in both secondary and latent syphilis patients

**Figure 4:**
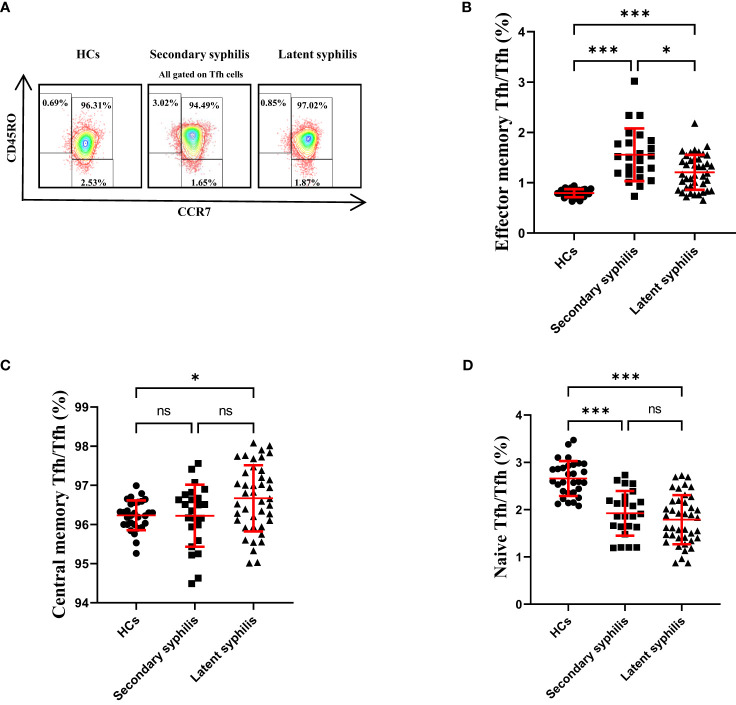
The percentage of effector memory Tfh cells in total Tfh cells showed a significant increase, whereas central memory and naïve Tfh cells did not exhibit significant changes. **(A)** Representative plots of CD45RO^+^CCR7^−^ effector memory, CD45RO^+^CCR7^+^ central memory, and CD45RO^−^CCR7^+^ naïve Tfh cell percentages in the peripheral blood of HCs (left), secondary syphilis patients (middle)and latent syphilis patients (right). The percentages of **(B)** effector memory, **(C)** central memory, and **(D)** naïve Tfh cells were compared between 30 HCs, 24 secondary syphilis patients, and 41 latent syphilis patients. *p<0.05, ***p<0.001, one-way ANOVA test. ns, significant.

Previously, CD45RO and CCR7 were used to define naïve and memory Tfh cells, which included CD45RO^−^CCR7^+^ naïve, CD45RO^+^CCR7^−^ effector memory, and CD45RO^+^CCR7^+^ central memory Tfh cells (21, 22). The central memory CXCR5^+^Tfh cells could support the antibody-mediated immune responses (23). Thus, we assessed these Tfh subsets in secondary syphilis and latent syphilis patients. Importantly, only the percentage of CD45RO^+^CCR7^−^ effector memory Tfh cells to total Tfh cells was significantly different among HCs secondary syphilis and latent syphilis patients (**Figures 4A, B**), indicating an association between these effector memory Tfh subsets and the clinical presentation of latent syphilis. In terms of the percentage of central memory Tfh cells to total Tfh cells, there was no significant difference between HCs and secondary syphilis patients. However, significant increase was observed between HCs and latent syphilis patients, also between secondary syphilis patients and latent syphilis patients(**Figure 4C**). With regard to naïve Tfh cells, while no significant differences were observed between secondary patients and latent syphilis patients. However, a significant decrease was observed both between HCs and secondary syphilis patients and between HCs and latent syphilis patients (**Figure 4D**).

### The percentage of Tfh2 cells was significantly different between secondary and latent syphilis patients

**Figure 5:**
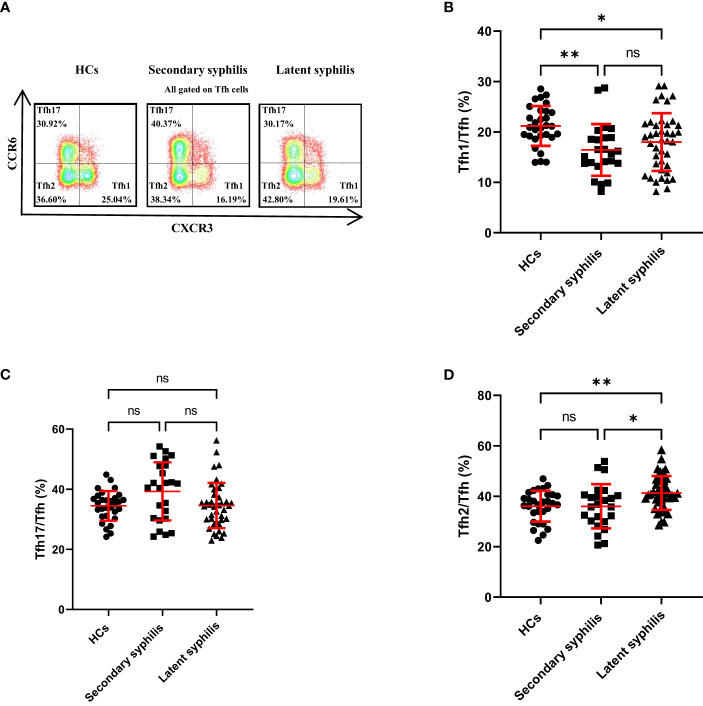
The percentage of Tfh1 cells in total Tfh cells was significantly changed between the secondary and latent syphilis patients compared to healthy controls while no significance was observed between secondary and latent syphilis. **(A)** Representative plots of Tfh1 (lower-right quadrant, CXCR3^+^CCR6^−^Tfh), Tfh2 (lower left quadrant, CXCR3^−^CCR6^−^Tfh), and Tfh17 (upper-left quadrant, CXCR3^−^CCR6^+^Tfh) cell percentages in the peripheral blood of HCs (left), syphilis patients (middle) and latent syphilis patients (right). **(B–D)** The percentages of Tfh1, Tfh2, and Tfh17cellsin total Tfh cells were compared between 30 HCs, 24 secondary syphilis patients, and 41 latent syphilis patients. *p<0.05 and **p<0.01, one-way ANOVA test. ns, not significant.

Morita (13) previously revealedthat human blood Tfh cells can be divided into (CXCR3^+^CCR6^−^Tfh) Tfh1, (CXCR3^−^CCR6^−^Tfh)Tfh2, and (CCR6^+^CXCR3^−^Tfh) Tfh17 subsets; only the Tfh2 and Tfh17 cell subsets could efficiently induce naïve B cells to produce immunoglobulins. Therefore, we wondered whether these Tfh subsets changed the pathogenesis of syphilis patients. Remarkably, the proportion of Tfh1 cells to total Tfh cells was significantly decreased in secondary and latent syphilis compared to HCs while no significant difference was displayed between secondary and latent syphilis patients (**Figures 5A, B**). In contrast, a significant difference in the proportion of Tfh17 cells to total Tfh cells was not observed between HCs and secondary syphilis patients, as well as between HCs and latent syphilis patients. However, a significant difference was observed between secondary syphilis patients and latent syphilis patients (**Figure 5C**). There was no significant difference between HCs and secondary syphilis patients for the percentage of Tfh2 cells to total Tfh cells, whereas significant differences were found between HCs and latent syphilis patients, as well as between secondary syphilis patients and latent syphilis patients (**Figure 5D**).

## Discussion

Penicillin is widely used for successful treatment of syphilis patients. Unlike other bacterial pathogens that quickly resist penicillin, *Tp* has remained sensitive (1). Syphilis infection can usually be divided into two groups: acute infection and latent infection. During acute infection, syphilis activates both innate and adaptive immune cells, whereas latent syphilis can effectively evade the immune system and persist in the host for more than 2 years in chronic latent infection (24). How *Tp* evades the immune response to cause persistence in the latent stage has not been fully elucidated (24). Recent evidence suggests that *Tp* may use the antigenic variation of bacterial surface proteins to evade the immune response (6). However, less attention has been paid to the immunological basis of syphilis pathogenesis at the cellular level, especially in latent syphilis patients (1, 2).

Animal and human studies suggest that the Th1 response is elicited in primary syphilis. A shift to a Th2 response is accompanied by progression to the secondary stage, allowing for incomplete clearance of *Tp* (7, 25–27). Tfh cells are a specialized CD4^+^ T cell subset that provides support to B cells in the germinal center for long-lasting humoral responses. Appropriate control of Tfh cell function is essential to human health, and overactivation can result in autoimmunity, while under activation is associated with immunodeficiency. In chronic virus infection, abnormal Tfh cells might also contribute to the observed B cell dysregulation and thereby delay the neutralizing antibody response. Moreover, reports highlighted that HIV replication could be concentrated within Tfh cells (28). Limited data also suggest that memory Tfh cells may play an important role in the subclinical or latent reservoir of HIV during antiretroviral therapy (28, 29). However, it is unknown whether the newly identified Tfh subsets play a role in the pathogenesis of syphilis.

Meanwhile, Tfh cells exhibit a multifaceted role in bacterial infections, contributing to antibody diversity, affinity maturation, and memory formation, thereby providing protective immunity against a wide range of bacterial pathogens (30). Contrary to viral infections where virus-specific Tfh cells assist in generating neutralizing antibodies (31), Tfh cells in bacterial infections facilitate effective humoral immune responses by interacting with B cells. When encountering bacterial pathogens, Tfh cells migrate to lymph node germinal centers and engage in interactions that promote the generation of high-affinity antibodies, supporting B cell differentiation into memory B cells and plasma cells (32). This process leads to the production of antibodies that neutralize bacteria, prevent their invasion, and aid in their clearance from the body (33). Additionally, Tfh cells play a crucial role in memory formation, enabling a rapid and effective immune response upon subsequent bacterial exposure (34). The interplay of Tfh cells with B cells and the production of antibodies contribute to the immune response against bacterial threats (35).

The immune response elicited during syphilis infection is a dynamic interplay between host defenses and the immune response to *Tp*. Our study delved into the role of Tfh cells, in the context of syphilis immunopathogenesis. Through an in-depth exploration of Tfh cell subsets’ distribution, phenotypic profiles, and activation status across different stages of syphilis, we provide novel insights into their potential contributions to disease progression and immune responses.

In this study, we revealed that the percentages of total Tfh cells significantly increased in secondary syphilis patients, indicating that Tfh cells may play an important role in the strong immune response of secondary syphilis. Furthermore, among Tfh1 cells, Tfh2 cells, Tfh17 cells,central memory Tfh cells, effector memory Tfh cells, effector Tfh cells, naïve Tfh cells, and resting Tfh cells, only the percentages of ICOS^+^Tfh or the effector memory Tfh cells significantly increased in secondary syphilis but decreased in latent syphilis patients. The identified decline in ICOS activation marker expression within Tfh cells might potentially link to immune responses during latent syphilis infection. Therefore, we hypothesized that in secondary syphilis infection patients, ICOS^+^Tfh or effector memory Tfh cells help B cells produce the neutralizing antibody to fight against syphilis, but in latent syphilis patients, these Tfh subsets do not activate the B cells to increase the production of neutralizing antibodies needed to eliminate syphilis from the human body. Therefore, whether these dysregulated Tfh subsets have protective abilities or play a pathogenic role in latent syphilis patients requires further investigation and validation by other laboratories.

This study acknowledges a significant limitation pertaining to the characterization of syphilis stages. Syphilis, a complex disease with distinct immunological features at each stage, manifests in multiple stages, namely primary, secondary, latent, and tertiary. Regrettably, our study primarily focused on the secondary and latent stages, lacking representation from the primary and tertiary stages. This limitation hinders a comprehensive evaluation of Tfh cells’ role across the complete spectrum of syphilis progression. The absence of primary and tertiary stage data impedes a thorough understanding of the dynamics and involvement of Tfh cells in evolving immune responses during different syphilis phases. Future research should address this gap by encompassing cohorts across all stages, facilitating a more in-depth assessment of Tfh cells and their implications in disease progression. Additionally, it is crucial to acknowledge other limitations of our study, including the relatively modest sample size and the focus on peripheral blood Tfh cells rather than those residing within lymphoid tissues. Further investigations involving larger cohorts and the integration of tissue-resident Tfh cells could provide a more comprehensive understanding of their roles in syphilis immunopathogenesis.

In conclusion, this study’s findings add to our understanding of the immune response against syphilis and highlight the potential significance of Tfh cells in different stages of the infection. The alterations observed in Tfh subsets provide insights into potential mechanisms of immune responses to *Tp*. Further research is needed to fully elucidate the roles of Tfh cells in syphilis and to explore the functional significance of these subset alterations.

Although highly effective penicillin treatment is available for syphilis patients, the best hope for the control of syphilis is the development of a vaccine that prevents both disease and transmission (6). Therefore, fully elucidating *Tp*’s human immune response or immune evasion mechanism is conducive to next-generation vaccine design. Thus, in this study, we highlight that these dysregulated Tfh subsets may play an important role in the pathogenesis of syphilis and can also be targeted for the next generation of syphilis eradication efforts.

## Data availability statement

The original contributions presented in the study are included in the article/**Supplementary Material**, further inquiries can be directed to the corresponding author/s.

## Ethics statement

The studies involving humans were approved by Zhejiang provincial people’s hospital. The studies were conducted in accordance with the local legislation and institutional requirements. Written informed consent for participation in this study was provided by the participants’ legal guardians/next of kin.

## Author contributions

FS: Writing – original draft. YS: Writing – original draft. YX: Formal Analysis. JZ: Formal Analysis. ZZ: Writing – review & editing. JL: Writing – review & editing. YG: Writing – review & editing. 
