# Supplementary figures and images for: Dysregulation of circulating T follicular helper cell subsets and their potential role in the pathogenesis of syphilis
Source: Front Immunol. 2023 Oct 12;14:1264508. doi: 10.3389/fimmu.2023.1264508 (PMC10600468; doi:10.3389/fimmu.2023.1264508)

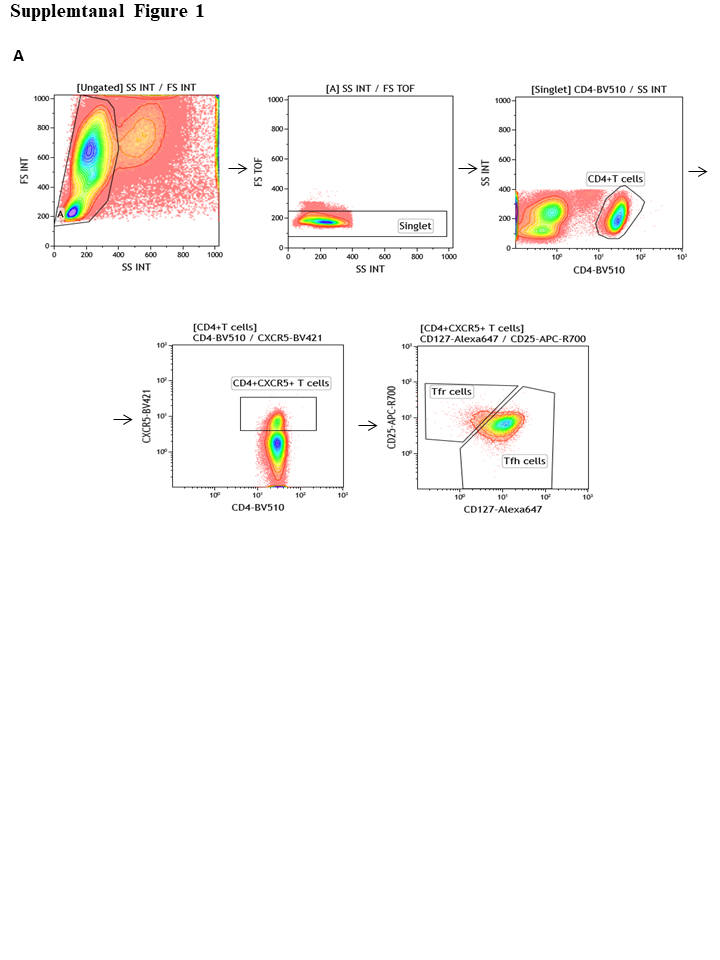

Supplement: Supplementary file 1 [file Image_1.png]
